# Supplementary material for: Sociodemographically differential patterns of chronic pain progression revealed by analyzing the all of us research program data
Source: PLOS Digit Health. 2025 Jun 17;4(6):e0000687. doi: 10.1371/journal.pdig.0000687 (PMC12173358; doi:10.1371/journal.pdig.0000687)
Supplement: Attachment — Submitted filename: Response_Reviewers_comments_v3.pdf [file pdig.0000687.s003.pdf]

Dear Anonymous Reviewers,

We appreciate your constructive comments. Your insightful feedback revealed that we had overlooked the multicollinearity between sex assigned at birth and gender, as well as the interactions between an initial condition and disparity factors. We have since corrected these issues, which have led to significant changes in our results. We apologize for any inconvenience caused by these unexpected changes and sincerely appreciate your reconsideration and further review of our revisions.

In this letter, responses appear in blue, additions in pink, and deletions are marked with strikethrough.

Sincerely,

Haiquan Li

Reviewers' Comments:

Reviewer's Responses to Questions

Comments to the Author

1. Does this manuscript meet PLOS Digital Health's publication criteria? Is the manuscript technically sound, and do the data support the conclusions? The manuscript must describe methodologically and ethically rigorous research with conclusions that are appropriately drawn based on the data presented.

Reviewer #1: Partly

Reviewer #2: Yes

Response: We have clarified our methods and results substantially.

2. Has the statistical analysis been performed appropriately and rigorously?

Reviewer #1: No

Reviewer #2: Yes

Response: We have revised our statistical model and updated the results accordingly.

3. Have the authors made all data underlying the findings in their manuscript fully available (please refer to the Data Availability Statement at the start of the manuscript PDF file)?

The PLOS Data policy requires authors to make all data underlying the findings described in their manuscript fully available without restriction, with rare exception. The data should be provided as part of the manuscript or its supporting information, or deposited to a public repository. For example, in addition to summary statistics, the data points behind means, medians and variance measures should be available. If there are restrictions on publicly sharing data—e.g. participant privacy or use of data from a third party—those must be specified.

Reviewer #1: No

Reviewer #2: No

Response: We are unable to share the original data per the policy of the All of Us Research Program, as it is controlled-tier data. However, we have shared our results fully in Supplementary File 1, which includes the insignificant ones.

4. Is the manuscript presented in an intelligible fashion and written in standard English?
PLOS Digital Health does not copyedit accepted manuscripts, so the language in submitted articles must be clear, correct, and unambiguous. Any typographical or grammatical errors should be corrected at revision, so please note any specific errors here.

Reviewer #1: No

Reviewer #2: Yes

Response: We have greatly expanded our manuscript and proofread our language to make it more intelligible.

5. Review Comments to the Author

Qi.1: Reviewer #1: This paper by Edwin Baldwin et al., titled "Incidence and Progression Patterns of Chronic Pain Disparities Revealed by Analyzing the All of Us Research Program Data" explores a valuable contribution to understanding the demographic disparities in the progression of chronic overlapping pain conditions (COPC) and associated mental health conditions.
One of the strengths of the study is the use of the All of Us Research Program dataset, which provides a large and diverse cohort that allows for a more nuanced examination of how sex, race, and ethnicity impact the progression of chronic pain and comorbid mental health disorders.

Ri.1: We are appreciative of your thoughtful review, insights, and comments. We have addressed your concerns (see below).

There are a few points that the authors should address to improve the paper:

1 Introduction
Qi.2 The introduction is well-written and sets the stage by addressing the broader context of disparities in chronic pain and mental health. The focus on underrepresented populations is both timely and highly relevant. However, this section could be enhanced by a deeper explanation of why certain demographic groups experience more severe progression of COPCs. Adding citations on factors like unique pain mechanisms, social determinants, or healthcare barriers specific to these populations could offer valuable context and strengthen the argument.

Ri.2: We appreciate your insight and have added more citations on unique pain mechanisms, social determinants, and healthcare barriers for underrepresented populations, as follows:

"Previous research reported a strong association between demographic background ~~(such as racial minority groups)~~ [5] and pain-related health outcomes [8], which implied unique pain

mechanisms across racial/ethnic groups [9-11] and sexes [12-14]. Besides recognized biological differences, social determinants are shown to affect the experience of chronic pains [15]. Evidence indicates that people with financial challenges encounter greater healthcare barriers in accessing pain management [16]. Further, in urban populations, the intersection of sociodemographic factors has been shown to create unique identities that impact pain experiences [5, 17]."

Qi.3 On the other hand, the concept of COPCs could be more clearly defined, as it currently lacks sufficient detail. Additionally, it would be helpful if the authors elaborated on the rationale behind selecting specific COPCs and mental health conditions.

Ri.3 We have more clearly defined COPC's as the ten chronic conditions which are recognized by the National Institutes of Health Pain Consortium as COPCs, in addition to justification for the mental health conditions included. The text is changed as follows:

"Chronic overlapping pain conditions (COPCs), defined as a subset of comorbid chronic pains that co-occur at higher-~~-~~than-~~-~~expected levels in the general population, are driven by shared mechanisms [1] and tend to be more common in females [1]~~, significantly impact lives worldwide~~. ~~In this study we focus on ten chronic pains currently recognized by T~~the National Institutes of Health Pain Consortium currently recognizes ten COPCs:~~, commonly referred to as COPCs in literature:~~ chronic low back pains (CLBP), migraine (MHA), irritable bowel syndrome (IBS), temporomandibular disorders (TMD), fibromyalgia (FM), chronic fatigue syndrome (CFS), chronic tension-type headache (CTTH), endometriosis (ENDO), vulvodynia (VVD), and urologic chronic pelvic pain syndrome (UCPPS). COPCs significantly affect individuals worldwide, interfering with daily activities, mental health, and overall quality of life. Given their frequent comorbidities with mental health conditions [2], our study includes the four most frequently studied conditions—~~They are closely related to mental health conditions including~~ anxiety, depression, bipolar disorder, and schizophrenia [2-4]"

2. Methods

Qi.4 The use of logistic regression for analysing COPC progression is appropriate, yet further details on the model's covariates would improve the reproducibility of your findings.

Ri.4 We have removed gender as a covariate and added interaction terms in the model. We have clarified the model's covariates as follows:

"The demographic variables assessed include sex, race, and ethnicity, for the known relevance in chronic pain [7, 12-14, 25-27]. Additional covariates were incorporated including birth year, household income level, and region of state of residence."

Qi.5 The study's use of the All of Us dataset offers a robust data source. However, addressing potential biases related to electronic health records (EHR) is essential—especially in underrepresented populations. Highlighting any limitations in the EHR's ability to capture pain-related outcomes or disparities could help contextualize the findings.

Ri.5 We acknowledge the limitations related to EHR as follows:

"The study and results rely heavily on electronic health records, which are known to have

potential biases and noises. The All of Us Research Program is known to be enriched with underrepresented populations and diverse health conditions, which could potentially affect the effect size of the results."

Ri.6 While the exclusion criteria for EHR quality issues are mentioned, additional details on the specific types of data quality issues (e.g., missing data, inconsistencies).

Ri.6 We have clarified the exclusion criteria as follows:

"Only individuals sharing EHR data were included, and those whose data showed apparent quality or power issues were excluded, such as problematic dates, ages (over 110 years old in any record), less than two clinical visits, and disagreement between reported sex and gender."

"We excluded the persons who had more than one COPC or mental condition within the clearance period, for which the order of the condition was difficult to establish."

Qi.7 While "disparity factors" are mentioned, the rationale for selecting these specific factors could be further developed. Adding references to supporting literature on the relevance of these factors for COPC and mental health disparities.

Ri.7 We have added references in the Method Sections to justify why we choose to study sex, racial, and ethnic disparity of COPCs, as follows:

"The experience of pain is not uniform but intricately shaped by the interplay of sociodemographic factors, including sex, gender, age, race, ethnicity, and financial status (e.g., income) [5, 6], resulting in disparities known to particularly affect subpopulation [9]. Previous research reported a strong association between demographic background ~~(such as racial minority groups)~~ [5]and pain-related health outcomes [8], which implied unique pain mechanisms~~,~~ across racial/ethnic groups [9-11] and sexes [12-14]. Besides recognized biological differences, social determinants are shown to affect the experience of chronic pains [15]. Evidence indicates that people with financial challenges encounter greater healthcare barriers in accessing pain management [16]."

Qi.8 Providing context on why certain reference levels (e.g., "White" for race) were chosen would clarify the model's baseline comparisons and reinforce the methodological rigor.

Ri.8 We used groups with socioeconomic advantages as reference levels. We added the following text:

"For sociodemographic reference in the statistical analysis, male is the reference sex, White persons make the reference race, non-Hispanic White is the reference ethnicity, and the highest income stratum is the reference income level."

Qi.8 The criteria for identifying disease progression between multiple COPC or mental health conditions could be more explicit. Specifying whether progression is tracked between distinct conditions (e.g., from anxiety to depression) or within subtypes.

Ri.8 We clarified our scope of study for progression to distinct conditions, rather than within subtypes of the same condition in the introduction section, as follows:

“The primary objective of the research is to discover statistically significant sociodemographic ~~disparity~~ factors associated with one of the ten COPC conditions and four ~~comorbid~~ mental health conditions. We sought to reveal the differential progression patterns among these distinct COPC and mental conditions that were ~~of special interest for their well-documented~~ known for their frequent comorbidities with COPCs [4, 19]”

Qi.9 Details on how income levels were categorized and whether adjustments were made for regional cost-of-living differences.

Ri.9 We have clarified income levels and added the region of the state of residence as a covariate to the model, to increase the power. All of Us does not provide more granularity of residence smaller than the state. The revision reads as follows in the Methods section:

“Variables studied include onset ~~incidence~~ of each COPC and mental condition, sex at birth, age, race, ethnicity, ~~and~~ household income level, and region of state residence (e.g., northeast).”

“Income levels—stratified as below $25k, $25-$50k, $50-$100k, $100-$150k, $150-$200k, and over $200k—served~~s~~ as~~is~~ a proxy for socioeconomic status. Missing income data (Skip or Prefer not to answer in questionnaire response) was treated as a separate category.”

4. Results:

Qi.10 The table 1 includes “Other or Unknown” categories for both sex and race, but there is no definition for what constitutes “Other” or “Unknown.” Since sex is generally defined as a biological classification based on physical and physiological traits (e.g., chromosomes, hormone levels, and anatomy), could you provide data to support this classification?

Ri.10. We clarified the definition of other options of sex, race, and ethnicity allowable participants to choose during the enrollment in the All of Us Research Program as the footnote of Table 1. We have excluded individuals with sexes other than male and female due to a lack of power for those groups.

The unknown race and other ethnicity read as follows:

“Unknown Race included: I prefer not to answer, none indicated, and skip. Others Ethnicity: none of these, prefer not to answer, and skip. Intersex and transgender persons were excluded due to insufficient statistical power.”

Qi.11 Alternatively, if the study uses this term in a broader, social context—referring to gender (a social construct encompassing roles, behaviors, and identities)—this distinction should be made clear.

Ri.11: All of Us provides the gender variable in a broader and social context. However, the majority of them are correlated with sex. This caused serious multicollinearity issues in the results of our initial submission, which diluted the signals of sex disparities in the baseline due to putting gender into the model. Since those with different sex and gender are in a small number, which often does not have sufficient power or a large enough cohort to report (>20) for most pairs of conditions, we had to exclude them from the study and will pursue it when sufficient data becomes available. We revised the manuscript as follows:

"We did not include gender, which is a broader social construct concept, due to its high correlation with biological sex and insufficient data to reach the required power."

Qi.12 The label "COPC Incidence" may be misleading, as the data appears to show the prevalence of each condition within the study cohort, rather than the incidence (rate of new cases over time). Adjusting this label to reflect "prevalence" would prevent misinterpretation of the data.

Ri.12. We studied the earliest onset of each condition, which is different from both incidence (number of new cases within a specific time period) and prevalence (number of cases over a specific time period), although it may be more similar to prevalence if the participants are still alive). So, we prefer to revise to the most actual meaning, onset, and replace all the occurrences of 'incidence' in the manuscript with 'onset'. The title has been revised as:

"**Sociodemographically Differential~~ Incidence and Progression~~ Patterns of Chronic Pain Progression~~Disparities~~ Revealed by Analyzing the All of Us Research Program Data**"

"**COPC Onset~~Incidence~~**" (Table 1)

"(3) Determining the~~ incidence, or~~ earliest onset: (…)" (Methods)

Qi.13 Please explain the criteria participants used for self-identification or how race and ethnicity data were gathered, as variations in options could lead to inconsistency in reporting. The "Other or Mixed" racial category comprises a relatively large portion (3.3%) but lacks specificity. Clarifying which racial or ethnic identities are included. Additionally, please specify if "Other or Mixed" refers to a blend of ethnic backgrounds or individuals identifying with multiple racial identities.

Ri.13. We clarified that sex at birth, race, ethnicity were self-identified. We have listed the specific racial groups in Table 1 and clarified the aggregated statistics for several occasions. We separated "Other or Mixed" back to their original category "More than One Population", "Middle Eastern or North African", and "None of these".

We have clarified the demographic definition as follows:

"Sex at birth, race, and ethnicity were self-reported during the enrollment of AoU." (Methods)

The following notes are added after Table 1:

"Unknown Race included: I prefer not to answer, none indicated, and skip. Others Ethnicity: none of these, prefer not to answer, and skip." (Results)

Qi.14 The term "non-White races" is somewhat vague (Table 2). Using more specific language, such as "racial and ethnic minorities," or explicitly listing racial groups (e.g., "Black or African American, Asian, Native Hawaiian or Other Pacific Islander").

Ri.14. Thanks for the suggestion. We have removed the term "non-White" in the manuscript and changed it to racial minorities in the tables and figures.

The manuscript is revised as follows:

"Table 2 demonstrates the elevated risk of specific COPCs in females and~~ in non-White ~~racial minoritiese~~s~~, based on multiple logistic regression studies of these variables using

demographically matched cohorts.”

Qi.15 The table includes categories such as “Middle Eastern or North African,” “More than One Population,” and others. If these categories were self-reported, providing definitions for each at the beginning of the study would be helpful, as some may overlap or be interpreted differently.

Ri.15 All of Us refer “Middle Eastern or North African” as individuals from the middle eastern or being a north African descendant. More than one population indicates those from descendants of multiple populations. We clarified those are self-reported and made a note under the table, as follows:

“Sex at birth, race, and ethnicity were self-reported during the enrollment of AoU.” (Methods)

“Of note, AoU includes a racial minority of Middle Eastern or North African for descendants of the region.” (Notes of Table 1)

Qi.16 To enhance readability, please provide a legend for clarify “ns” as “non-significant, p > 0.05.” ”insignificant” does not meet the acronymic nomination criteria.

Ri.16. We clarified under Table 2 and clarified those are p-values after Bonferroni correction.

“*Note*: OR: odds ratio. ns: not ~~in~~significant after Bonferroni correction, adj.p>0.05, *: 10^{-3}≤adj.p<0.05, **: 10^{-6}≤adj.p<10^{-3}, -***: adj.p<10^{-6}.”

Qi.17 While endometriosis (ENDO) and vulvodynia (VVD) are appropriately noted as “exclusive” to females, a note explaining these as conditions specific to individuals with female reproductive anatomy would provide helpful context for those less familiar with these terms. Consider not include in the Table this information.
The terms “non-White races” and “reference demographics” (White, non-Hispanic, male) could benefit from more precise language.

Ri.17 We removed endometriosis and vulvodynia in Table 2 and noted that they are female-specific and no identified disparity in racial and ethnic minority groups as well. It is revised as follows:

“Since no significant racial or ethnic disparity factors have been identified for endometriosis and vulvodynia—female-specific disorders—they are not shown in the table.”

We also clarified non-white races in Ri.14 and clarified the reference demographics as follows:

“For sociodemographic reference in the statistical analysis, male is the reference sex, White persons make the reference race, non-Hispanic White is the reference ethnicity, and the highest income stratum is the reference income level.” (Figure 1 legend)

Qi.18 Clarify the “solid edges with hollow triangle arrows” by noting their significance, such as “solid edges indicate a strong association,” which would enhance visual understanding. While color coding by demographic group is effective, providing a legend specifying which color represents each group (e.g., “Black arrows for Black individuals, orange for Hispanic individuals”).

Ri.18. We have redesigned the visual schema and put a legend beside the figure, as well as briefly described in the captions. It reads as follows:

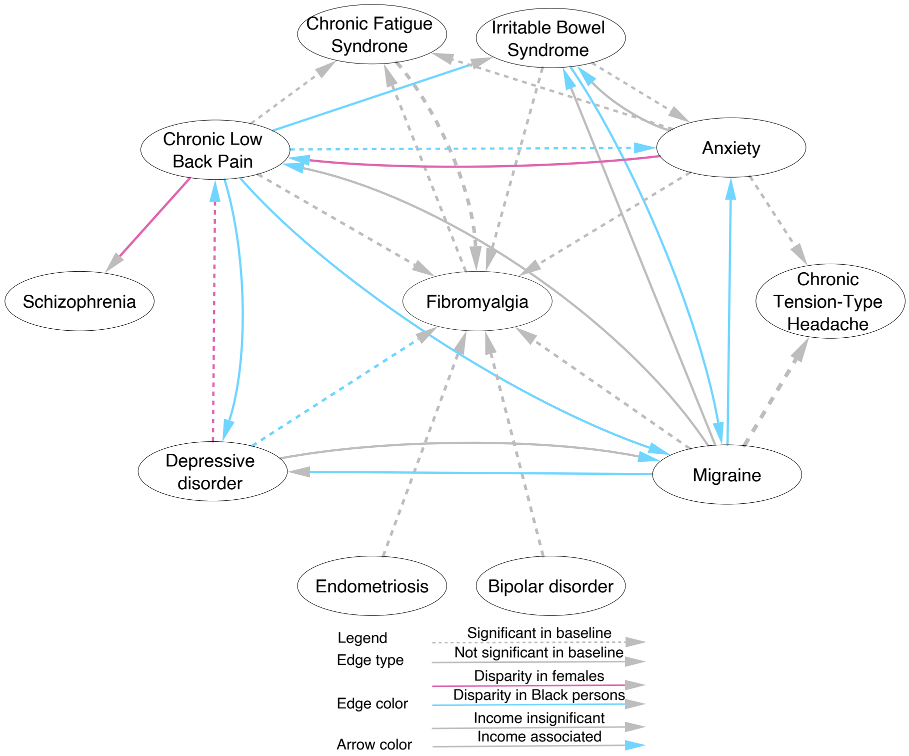

"**Fig 1. The overrepresentation of specific pairwise relations between COPC and mental conditions, comparing females, ~~and non-White~~ racial minority persons, and persons with low income to the reference sociodemographic groups**. Each node represents either a specific COPC condition or a mental disorder. For sociodemographic reference in the statistical analysis, male is the reference sex, White persons make the reference race, non-Hispanic White is the reference ethnicity, and the highest income stratum is the reference income level. Each a~~A~~rrowed edge depicts a significant temporally ordered disease pair supported by either the ~~occurrences of conditions based on~~ reference demographics (~~white, non-Hispanic, or male~~ dashed edges) or the alternative demographics (solid edges). Edge colors ~~Solid edges with hollow triangle arrows~~ indicate significant (Bonferroni-correlated p-value<0.05) association with specific ~~further enrichment for specific~~ demographics ~~demographic subgroups that are color-coded~~ (gray for White, blue for Black or African American, and purple for females), while arrow colors indicate significant overrepresentation of the lowest household income levels in contrast to the highest level (gray for none and blue for significant).~~: each Black arrow indicates that for the specific directed pair of conditions Black or African American persons have significantly higher risk in comparison to White persons. Likewise, orange arrows for Hispanic or Latino persons as compared to non-Hispanic persons, purple arrows for females compared to males, and dark purple arrows as females vs. males or African Americans vs. Whites.~~"

Qi.19 To improve comprehension, a brief introductory explanation of the node and connection

symbols would be helpful before diving into the detailed analysis. This will make the visual representation more accessible to readers unfamiliar with these symbols.

Ri.19. We added some nodes and connection descriptions before discussing the disparity pattern in Fig. 1, as follows:

"Each arrow~~ The connections~~ between two nodes indicates ~~how strongly ~~the twose conditions are associated sequentially ~~with each other in sequence, ~~accordingly~~ by~~ using regression analyses using non-Hispanic White males as the reference group."

Qi.20 Including confidence intervals alongside the odds ratios.

Ri.20. We included confidence intervals alongside the odds ratios in all occurrences of the manuscript, such as:

"Females present higher odds ratios of developing anxiety and depression at 1.50 (95% CI [1.44, 1.56])~~1.39~~ and 1.48 (95% CI [1.42, 1.54])~~1.33~~ with adjusted p-values less than ~~of 1.13 x~~10^{-16}~~^{3}~~ ~~and 1.19 x10^{-2} ~~."

4. Discussion:

Qi.21 The discussion would benefit from further comparison with previous studies that have examined disparities in single COPCs. While the authors highlight the novelty of their approach in studying progression disparities, further contextualization with similar research could help underscore the impact of this study.

Ri.21: We have significantly expanded this section, comparing our disparity results in single COPCs with previous studies, which have high consistency. While our results identified known disparities in the baseline, such as enrichment of COPCs in females and chronic low back pain in Black or African Americans, our results highlight the elevated risk for individuals with an existing condition, many of which were not reported before. We revised the manuscript as follows:

"Our results align with ~~recaptured previous evidence ~~known~~ on the~~ disparities in developing of COPC and their comorbidities~~ incidence~~,~~which may be attributed to subconscious bias within the health system.~~ e.g., the elevated risk for females to develop all COPCs [28, 29], except UCPPS, bipolar disorder, and schizophrenia [30]."

"For instance, our findings indicate that females are at a significantly heightened risk for developing chronic low back pain[33] following an anxiety or depression diagnosis. Although the impact of psychological conditions on chronic low back pain has been widely studied [32-36], the influence and magnitude of sex differences in the progression from a mental disorder to CLBP remain inconclusive. These findings offer new insights into this issue."

"The results indicated an increased risk of developing chronic low back pain (CLBP) in Black or African Americans [17], who may also face a higher risk for subsequent COPCs (e.g., irritable syndrome) and mental conditions (e.g., anxiety and depression). Although CLBP is more prevalent among African Americans, its differential influence on other COPC pains or mental conditions has been rarely reported in Black Americans, particularly by longitudinal and prospective studies [37]. A few studies suggested Black population with chronic pains~~,~~ reported more depressive symptoms [38]."

Qi. 22 A section on potential mechanisms driving the observed disparities would add depth to the discussion. For instance, expanding on biological, social, and psychological explanations for increased pain susceptibility or mental health conditions in certain populations (such as higher inflammation rates in females or the role of socioeconomic stressors in marginalised groups) could provide valuable insights.

Ri. 22. We appreciate the recommendations. We added a brief, but informative section on potential mechanisms that drive the disparities such as sex differences in biological mechanisms, socioeconomic influence on pain management, and psychological stress from the communities, increasing mental burden. Our revisions include:

"Possible explanations may involve biological differences between sexes [13], such as hormone factors [28], sensitization [17], and inflammatory responses [25]."

"This may be related to the racial differences in pain sensitivity and perception [26, 39-41], psychological response to chronic pain [42], stress levels [38], and allostatic load [43, 44]. Of note, socioeconomic confounders cannot be ruled out in the context of healthcare access and utilization [31, 45, 46], which would influence the management and therefore containment of disease progression."

Reviewer #2: Comments:

Qii.1 This study investigated the disparities in progressions in developing COPCs and mental conditions. The findings of this study added an important contribution to understand the interactions of COPCs and mental conditions among underrepresented populations and develop effective COPCs management strategies.

Rii. 1. We appreciate your positive comments.

Abstract:

Qii.2. Lines 24-26. Why reported subsequent depression after irritable bowel syndrome as an example in abstract? Because the disparities in this progression pathway is the most significant one? Or any other reasons? As a reader, I would also wonder what the nine progression pathways are. I understand that the word count is limited for the abstract, any possible ways to mentioned two or three out of the nine?

Find a solution to summary that.

Rii.2 We initially could only make room for one and chose the most statistically significant to include in the abstract. We have summarized the results of disparity in COPC/mental disorder progression in the abstract. It reads as follows now:

"Black or African Americans with a COPC condition had a significantly increased association in progression to other COPCs (CLBP->IBS, CLBP->MHA, or IBS->MHA, $OR \geq 1.25$, $adj.p \leq 4.0x10^{-3}$) or mental disorders (CLBP->anxiety, CLBP->depression, MHA->anxiety, MHA->depression, $OR \geq 1.25$, $adj.p \leq 1.9x10^{-2}$) after developing a COPC. Females had an increased likelihood of chronic low back pain after anxiety and depression ($OR \geq 1.12$, $adj.p \leq 1.5x10^{-2}$). Additionally, the lowest income bracket was associated with an increased risk of developing another COPC from a COPC (CLBP->MHA, IBS->MHA, MHA->CLBP, or MHA->IBS, $OR \geq 1.44$, $adj.p \leq 2.6x10^{-2}$) or from a mental

disorder (depression->MHA, depression->CLBP, anxiety->CLBP, or anxiety->IBS, $OR \geq 1.50$, $adj.p \leq 2.0 \times 10^{-2}$), as well as developing a mental disorder after a COPC (CLBP->depression, CBLP->anxiety, MHA->anxiety, $OR \geq 1.37$, $adj.p \leq 1.6 \times 10^{-2}$)."

Introduction

Major comment:

Qii.3 The second half of the introduction are mostly about the study's objectives but rarely study innovation. Lines 51-55, you mentioned that previous evidence in disparities in the progressions of COPCs are limited. Why you think this is an important question? I see that you only mentioned in lines 58-59 that they "were of special interest for their well-documented interactions with COPCs". Can you add more justification for the study in the introduction?

Rii.3: We appreciate the critical question you raised. We have clarified the significance of the study and justify the rationale as follows:

"Validation of such differential disease manifestations will allow for more precise prevention strategies that target the relative risk of further COPC complications in specific subpopulations."

Minor comment:

Qii.4. Lines 44-45, "including anxiety, depression, bipolar disorder, and schizophrenia" are the four comorbid mental health conditions mentioned in line 57, right? If yes, would add "four" before "mental health conditions" in line 44, as. You did for the ten chronic pains to help readers quickly link them together.

Rii.4: We appreciate the comment on consistency and have added the descriptor as well as further citations justifying the connection between COPC's and the four mental health conditions. We revised the manuscript as follows:

"Given their frequent comorbidities with mental health conditions [2], our study includes the four most frequently studied conditions—~~They are closely related to mental health conditions including~~ anxiety, depression, bipolar disorder, and schizophrenia [2-4]."

Results:

Minor comments:

Qii.5. Line 67, what is AoURP v7 dataset? Version 7 or specific survey year? Please clarify.

Rii.5. It meant AoURP version 7 dataset. We have updated the results to Version 8 which was updated recently. We have defined it in the first occurrence as follows:

"Table 1 shows the demographic characteristics of the COPC cases and their comorbid mental disorders in the AoURP version 8~~7~~ dataset."

Qii.6. Table 1, any missing cases? How are they excluded?

Rii.6. Our analysis was based on EHR in the AoU biobank. Participants with EHR are labelled in the system. As the disorders are chronic conditions, the chance of missing the whole condition is unlikely. However, it is possible some episodes, including the first onset might be missing due to the incomplete information the participants provided. We clarified what was excluded in

methods as follows:

"Only individuals sharing EHR data were included, and those whose data showed apparent quality or power issues were excluded, such as problematic dates, ages (over 110 years old in any record), less than two clinical visits, and disagreement between reported sex and gender."

We also acknowledge incomplete information in the discussion:

"The study and results rely heavily on electronic health records, which are known to have potential biases and noises."

Qii.7. Lines 72-84, and the entire results section, sometime p value is exactly reported (e.g. CLBP, p<9.18*10-15), sometime p value is reported in a format of p<10-10 (line 83). Please keep using a consistent format. Suggest the later way.

Rii. 7. We have kept it consistent by truncating p-values if they are less than 10^{-16}, which will become less precise in the R system. In addition, all p-values have been accompanied by confidence intervals, both of which were adjusted by Bonferroni correction. For instance:

"For schizophrenia, a significant odds ratio of 2.61~~2.16~~ (95%CI [1.93, 3.52], adj.p$<10^{-16}$~~0~~) was observed in Black or African American persons, which corroborates the literature [21, 22]."

Qii.8. Fig.1 This is a nice figure summarizing the findings of the interactions between COPC and mental conditions. Suggest adding corner labels/legends to clarify black arrows indicates significant disparities among Black persons, orange arrows indicates Hispanic/Latino, etc. I see they are described as texts in lines 95-98. Corner labels/legends will complete the figure so that readers can quickly catch the key findings.

Rii.8 We appreciate the comments. We have updated the figures based on the updated results, added legends within the figure, as shown below:

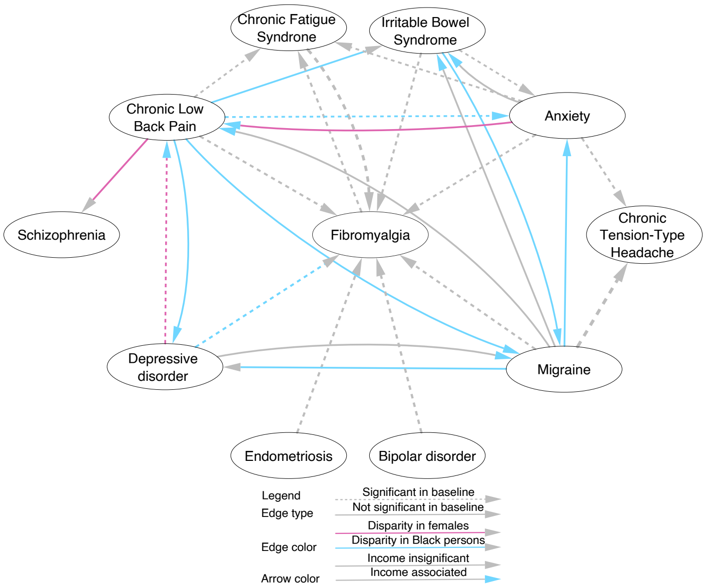

We also updated the caption of the figure as follows:

“**Fig 1. The overrepresentation of specific pairwise relations between COPC and mental conditions, comparing females, racial minority persons, and persons with low income to the reference sociodemographic groups**. Each node represents either a specific COPC condition or a mental disorder. For sociodemographic reference in the statistical analysis, male is the reference sex, White persons make the reference race, and the highest income stratum is the reference income level. Each arrowed edge depicts a significant temporally ordered disease pair supported by either the reference demographics (dashed edges) or the alternative demographics (solid edges). Edge colors indicate significant (Bonferroni-correlated p-value<0.05) association with specific demographics (gray for White, blue for Black or African American, and purple for females), while arrow colors indicate significant overrepresentation of the lowest household income levels in contrast to the highest level (gray for none and blue for significant). ”

Methods:
Minor comments:
Qii.9. 127, can you clearly define/list your reference group. I read that it is male for sex, non-Hispanic for ethnicity, White for race in results section. But I think you need define them clearly in methods first, and why they are selected as the reference group?

Rii. 9: Thank you for pointing out the issue. We used groups with socioeconomic advantages as reference levels and have modified the text to indicate the reference level for each factor. The text now reads as follows:

“For sociodemographic reference in the statistical analysis, male is the reference sex, White persons make the reference race, non-Hispanic White is the reference ethnicity, and the highest income stratum is the reference income level.”
